# Intelligent Evaluation Method for Design Education and Comparison Research between visualizing Heat-Maps of Class Activation and Eye-Movement

**DOI:** 10.16910/jemr.17.2.1

**Published:** 2024-10-10

**Authors:** Jia Jiayi, Zhao Tianjiao, Yang Junyu, Wang Qian

**Affiliations:** Tianjin University, Tianjin, China

**Keywords:** Eye movement, Eye tracking, Gaze, Attention, Design education, Design evaluation, Deep learning

## Abstract

The evaluation of design results plays a crucial role in the development of design. This study presents a design work evaluation system for design education that assists design instructors in conducting objective evaluations.

An automatic design evaluation model based on convolutional neural networks has been established, which enables intelligent evaluation of student design works. During the evaluation process, the CAM is obtained. Simultaneously, an eye-tracking experiment was designed to collect gaze data and generate eye-tracking heat maps. By comparing the heat maps with CAM, an attempt was made to explore the correlation between the focus of the evaluation’s attention on human design evaluation and the CNN intelligent evaluation. The experimental results indicate that there is some certain correlation between humans and CNN in terms of the key points they focus on when conducting an evaluation. However, there are significant differences in background observation.

The research results demonstrate that the intelligent evaluation model of CNN can automatically evaluate product design works and effectively classify and predict design product images. The comparison shows a correlation between artificial intelligence and the subjective evaluation of human eyes in evaluation strategy. Introducing artificial intelligence into the field of design evaluation for education has a strong potential to promote the development of design education.

## Introduction

As society continues to develop, the public is placing greater emphasis on their quality of life and user experience. This has led to an increased demand for innovative product design, and universities have become key in the cultivation of design talent. To optimize design quality, it is crucial to conduct research and improve design education within universities, as they are essential for cultivating design talent and meeting the increasing demand for innovative design.

In design education, creativity is a closed loop consisting of knowledge, imagination, and evaluation. The evaluation of design results is the last stage of design education and serves two main purposes. First, it assesses the quality of design outcomes and guides design decision-making. Second, it intervenes in the design process and indicates the direction for the iterative optimization of designs. There are two main types of design evaluation methods. (1) Human-based evaluation methods include the creative product semantic scale (CPSS) (1; 29), based on Teresa Amabile's consensus evaluation technology and the creativity support index (CSI) (4). (2) Computer-based evaluation methods include methods that rely on prior knowledge and use machine learning for feature training (8). By defining color features and social attributes of an image, visual quality, popularity, and other qualities can be predicted(18).

Both methods for evaluating design have their limitations. For human-based evaluation methods, creating scoring criteria and providing training to raters is a time-consuming and laborious process that demands significant effort. Moreover, design coursework is often displayed as renderings or hand-painted images, making design evaluation primarily image-based. The traditional evaluation of product design in education has issues such as non-uniform indicators and subjective scoring. At the same time, when students receive performance feedback, they often receive a single total score, which does not help target improvement in areas that need improvement. Computer-based evaluation methods typically only provide evaluation results, making it difficult to receive and understand non-verbal feedback. Therefore, in design education, a smart evaluation mechanism is needed to integrate knowledge, experience, and computing advantages; serve design students; and enhance their abilities.

The rise of the Internet has made it easier for people to access image data. With the continuous advancement of artificial intelligence, computers can recognize implicit relationships among images that are not visible to the human eye. This enables the intelligent classification and evaluation of images. Advancements in technologies such as image recognition provide a strong opportunity in design education. With the objective and efficient evaluation of design images, it is possible to improve the quality of design education.

This study applies deep learning to design image data, combining big data computing power and artificial experience to develop intelligent evaluation methods for design education. First, a vast assortment of award-winning images was used as the input. Then, using convolutional neural network (CNN) automatic feature extraction, a multi-task weakly supervised model for evaluating product design images was built. Finally, by comparing the evaluation heat map of eye movement data with CNN's class activation map (CAM), the effectiveness and interpretability of intelligent evaluation are verified, and efficient and reliable design job evaluation is finally realized.

### Design Evaluation Research

Traditional design evaluation typically relies on subjective human scoring, and its related research focuses on the establishment of a rating system and increasing the objectivity of the evaluation. Li et al. used an improved analytic hierarchy process (AHP) to evaluate industrial design (22). Cao et al. developed an evaluation model using a fuzzy mathematics-based synthetic evaluation model and AHP to identify factors that affect the of evaluation color comfort (3). Wang et al. researched eye-tracking technology and product a design evaluation that summarizes a set of evaluation processes for product design (40). Wang et al. used a multi-modal strategy that fuses electroencephalography and eye movement to investigate design decision-making and found that physiological signals can reflect subjective evaluation (39). Kim et al. created universal design evaluation indicators for mobile phones to analyze whether they meet universal design requirements (19).Because of the uncertain nature of design, an increasing number of researchers favor the fuzzy comprehension evaluation (FCE) method (24; 26). Chan et al. developed an evaluation method to assess the environmental performance of product design throughout its life cycle using life cycle assessment and a fuzzy AHP (6). Moreover, design evaluation has benefited from virtual reality technology, which enables comprehensive observation and evaluation (30; 44).

It is undeniable that human-based evaluation possesses an unparalleled significance and demonstrates remarkable flexibility, especially when dealing with intricate assessments. Nonetheless, in comparison to advanced intelligent evaluation methodologies, human-based evaluation methods are prone to subjective influences stemming from the evaluator. Additionally, when tasked with evaluating a substantial corpus of works, human evaluation often comparatively less efficient.

### Research on Intelligent Design Evaluation

With the advancement of artificial intelligence, there is a growing interest in automatic evaluation models for design, resulting in numerous emerging algorithms. Huang et al. developed an approach using computational intelligence for product concept generation and evaluation (14). Gao et al. employed the Delphi technique method, fuzzy comprehensive evaluation method, and gray comprehensive evaluation method for the quantitative evaluation of product design plans (11). Tsai et al. established two evaluation models. Model I uses fuzzy neural networks to predict the overall image, whereas Model II uses gray clustering for color image evaluation and two fuzzy neural networks for formal and overall image evaluation (36). Dou et al. used deep neural networks to extract representative features from web pages, quantifying their aesthetics. They proposed an automated method for calculating web page aesthetics based on deep learning techniques (9).

Compared with the model mentioned above, VGG16 has a deeper network and smaller convolution kernel (34), and hence it can extract more feature information during image processing, reduce the number of parameters, reduce the risk of over-fitting, and ultimately improve the accuracy and efficiency of image processing. Therefore, we selected a VGG architecture to build the intelligent design evaluation model.

Few research studies have used design assignments as evaluation data sets in design education, and the majority of current intelligent design evaluation methods are aimed at relatively mature design products.

### Research on Eye Movement and Design

Eye tracking technology uses computers and cameras to process human eye movements. Through technological means, it digitizes and visualizes human eye data, enabling the tracking and analysis of eye movement trajectories, thereby revealing the cognitive and behavioral processes of the subject. This technology has the advantages of high accuracy, low delay, and non-invasive measurement. Initially used in reading research, it has been increasingly applied in sociology, psychology, human–computer interaction, and other research fields.

In the field of education, some researchers have used eye movement data in their studies. Halszka Jarodzka team introduced the application of eye tracking technology in three fields of educational science: Instructional Design, expertise development, and eye movement modeling examples, and listed the main educational theories. They pointed out that this is a new research field that requires more research to expand (17). Leen Catrysse and others found through eye-tracking data that different backgrounds in educational videos will have an impact on students' attention in the process of watching videos, and neutral backgrounds will better keep students' attention (5).

Because of its high correlation with vision, eye tracking technology has become an important tool in studies of the human visual system on topics such as visual attention, visual search, and visual processing. It is often used to evaluate the effect of design on visual interfaces and products. Through the analysis of eye movement signals, we can determine the eye movement index for an experimental object in the subjective evaluation of its design, and then reveal the cognitive processes and thinking mode of humans. Chun Cheng Hsu et al. chose 16 chairs with different shapes as an evaluation object, and through the analysis of eye movement data, found that participants usually focused on two parts of the chair (the seat and backrest), when making a perceptual evaluation, which showed that the seat and backrest were the two main features that people considered when evaluating a chair. They also proved that we can predict a person’s perceptual evaluation of product shape by analyzing their eye movement (13). Peng Liu et al. developed an evaluation method of product appearance design based on eye tracking and aesthetic measurement. Taking the design of campus street lamps as an example, eye-tracking technology was used to evaluate the aesthetic feeling of each scheme, and the best design scheme was obtained (25).

In addition to three-dimensional product appearance evaluation, eye movement research is also frequently used in two-dimensional interface evaluation and color scheme evaluation in the field of design evaluation. Hongxia Li et al. used an eye-tracking method, heat map, an eye movement availability index, information processing efficiency, and pupil size to represent overall effect, efficiency, physical satisfaction, and other indicators. They then built an evaluation model for a coal machine mechanical interface based on eye movement experimental data (20). The research of Yong Wang et al. shows that a color scheme compensatory evaluation method based on eye movement tracking can effectively evaluate the color scheme of a product design and provide a reference for the evaluation and decision-making of color scheme designers (38). Yixiang Wu used eye movement data such as viewing time and fixation points to evaluate the usability of a smartwatch interface (41).

Moreover, using the research results of eye movement physiological signals, product design can be optimized and improved. Through two eye-tracking experiments, Niu Ya Feng et al. obtained that the optimal control size of ECI interaction is 256 × 256px2 and the optimal dwell time is 600ms, which provides a theoretical basis for the improvement of ECI interaction interface design (28). Yavuz Inal designed an eye movement experiment in which 32 participants (16). observed the position of error messages in Web Forms in four different web forms and captured the subjects' eye movement tracks. Through the analysis of eye movement data, it is found that when the error message is displayed on the right side of the error input field, participants will find the error message fastest, which will promote the placement of error messages in web design. Mu Tong et al. found that the complexity of rotating elements will affect participants' subjective perception of speed, to guide the design of rotating elements in human-machine interfaces (35).In the field of education, scholars also use eye movement data to explore.

Although eye-tracking technology is widely used at present, it is more concentrated in the field of simple design, and research in the field of design education is rare.

## Research Questions and Procedure

### Research Questions

In this paper, the researchers investigated three questions.

Question 1: What are the criteria used to evaluate design education, and how are the weights of each evaluation index allocated?

Question 2: How can we create an intelligent evaluation model using the current evaluation data?

Question 3: What is it that humans and artificial intelligence focus on in a design evaluation? Moreover, what is their correlation?

### Research Procedures

**Figure 1.**
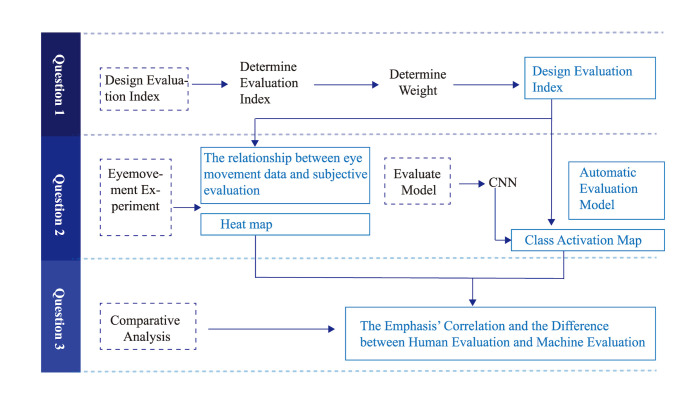
Overall Research Framework

The three research questions of this study were comprehensively investigated in the three parts of this study. In the first part, the researchers analyzed evaluation standards in design education, and established indicators and weights of design evaluation using factor analysis and AHP. For the second part, using the design evaluation indicators obtained in part 1, relevant design data were gathered from a variety of sources. These data served as the foundation for creating an intelligent evaluation model. In this study, the researchers developed an automatic design evaluation model using a CNN and obtained its CAM. The researchers studied the third question by comparing and analyzing the eye movement heat maps and CAM of the manual evaluation, that is, where do humans and artificial intelligence focus when conducting design evaluation, and what is their correlation? This is used to investigate the interpretability of intelligent evaluation models. There is a strong correlation and continuity between the various issues in this study, which are interdependent and explanatory of each other. The research framework is illustrated in Figure 1.

Procedure for determining the evaluation indicators: In this study, the design evaluation indicators for design education were determined by a series of analytical steps.

The first step was to conduct an extensive literature review to identify design evaluation indicators. These indicators were then sorted based on the frequency of their appearance in the literature, and the top indicators were selected for further analysis. In addition, interviews were conducted with teachers in the design education field to further refine the selection of indicators. Finally, the indicators applicable to student homework evaluation were obtained.

Next, online questionnaires were used to collect data, and dimensional-reduction analysis of the evaluation indicators was carried out using factor analysis to obtain the candidate design evaluation indicators.

**Figure 2.**
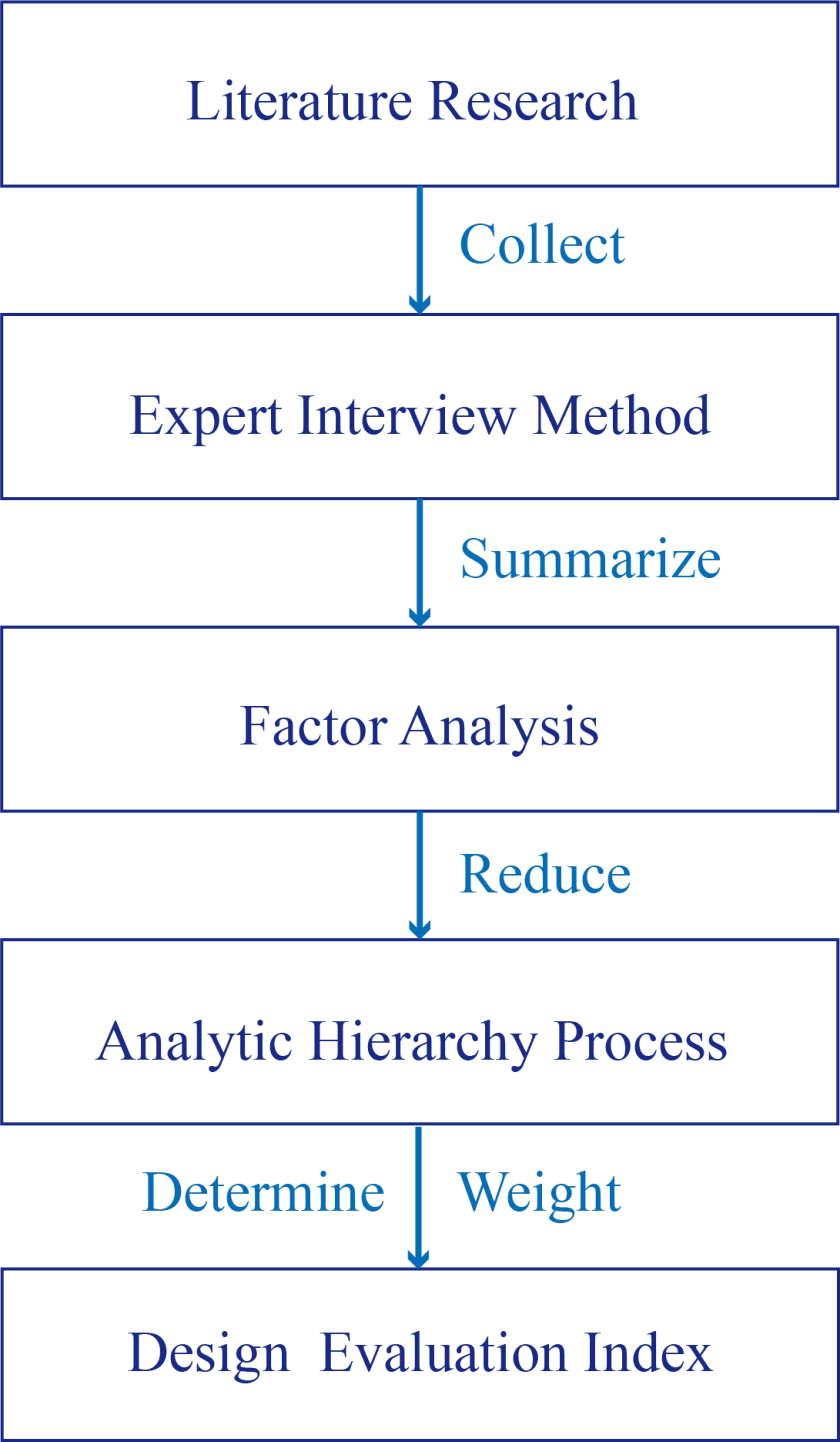
Framework for the Design Evaluation Index Research.

Finally, using the factor analysis results, the online questionnaire was reissued, and AHP was used to assign weights to each indicator for accurate design evaluation system standards in design education. Figure 2 depicts the framework for the design evaluation index research.

Procedure for Establishing the Design Evaluation Model: In this part of the study, we focused on developing a smart evaluation model for design education. To begin with, we collected a significant number of labeled data for the evaluation and created a data set for evaluating design education coursework. Using this data set, we established an automated design evaluation model using the CNN, generated the CAM, and conducted a comparative analysis in question 3.

**Figure 3.**
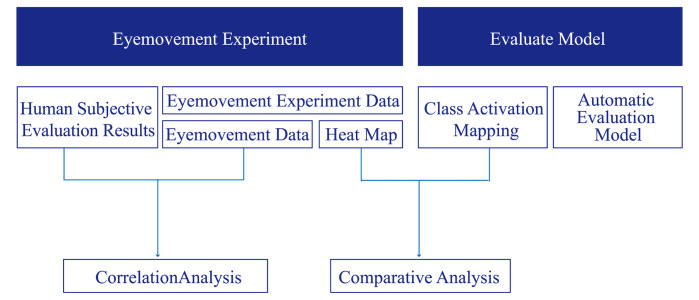
Framework for Determining the Correlation between Human and Artificial Intelligence Evaluation Methods.

Procedure for Determining the Correlation between Human and Artificial Intelligence Evaluation Methods: The research steps for this part are shown in Figure 3.

First, based on the findings of question 1, we conducted an eye movement experiment and obtained both subjective evaluations of the design and eye movement signal data from the participants. We then analyzed the relationship between the physiological signals and subjective evaluations.

Then, we analyzed the focus of the machine learning-based evaluation using CAM.

Finally, the eye-tracking heat map and CAM were compared to analyze their similarities and differences.

## Methods

During the research process, a mix of qualitative and quantitative research methodologies were used. The researchers involved in the research process included design teachers, designers, design students, and other people with a certain design experience. Selecting this group of individuals allowed for a more professional analysis of existing issues within design education.

### Expert Interview

In this study, design teachers in colleges and universities were interviewed using semi-structured expert interviews. The aim was to identify and evaluate design evaluation indicators. The interview questions focused on primary and secondary evaluation indicators, their ranking, and the weights assigned to them during design evaluations.

### Likert Scale

Likert-scale scoring was used for factor analysis and eye tracking.

During the factor analysis stage, we designed 10 questions to determine the importance of each of the 10 design indicators that were summarized in the literature research and interviews. A scale of 11 points was used for scoring and data collection, ranging from 0 (very unimportant) to 10 (very important).

During eye movement data collection, subjective evaluations of product design images were collected on a 7-point scale ranging from −3 (very bad) to 3 (very good).

### Factor Analysis

In this study, factor analysis was used to perform dimensional-reduction analysis on the evaluation indicators.

The specific steps of factor analysis are i) standardization of the data, ii) calculation of the data’s correlation coefficient matrix, iii) analysis of the correlation between variables, iv) calculation of the initial common factor and factor loading matrix, v) rotation of the factors, and vi) calculation of the factor score. In this study, the principal component method was used to estimate the factor loading in the process of calculating the initial common factor and factor loading matrix. The number of principal components is determined to determine the number of common factors. When determining the number of principal components, 85% were selected to ensure the chosen common factor reflects the overall information as much as possible. In this study, varimax was used during the rotation of factors.

### Analytic Hierarchy Process(AHP)

The steps of AHP include i) determination of the indicator system and establishment of the hierarchical evaluation model, ii) construction of the judgment matrix, iii) calculation of the weight vector, and iv) analysis of the result. In this study, the AHP method was primarily used to calculate the weight vector.

When constructing a hierarchical evaluation model, it is necessary to clarify the goal layer, criteria layer, and alternative layer.

**Figure 4.**
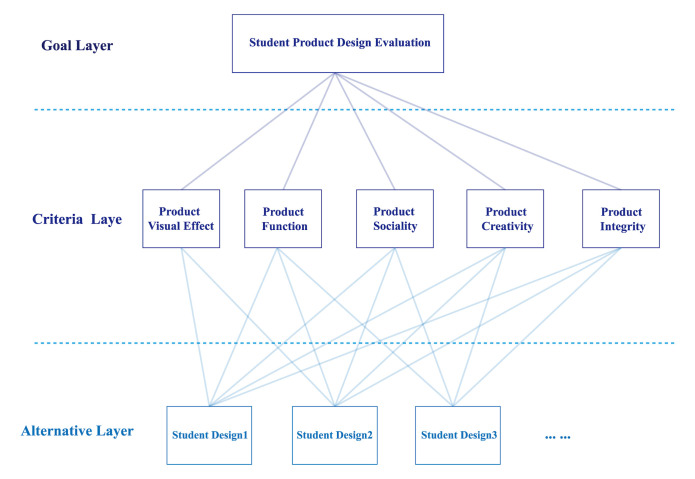
Hierarchical Evaluation Model

In this study, the goal layer is the evaluation of the students’ product coursework; the criteria layer is the result of dimensionality reduction analysis of five factors (product visual effect, product function, product sociality, product creativity, and design integrity); and the alternative layer is a specific student’s design plans. The hierarchical evaluation model of this study is shown in Figure 4.

### Eye-Tracking

Eye-tracking technology was used in this study to conduct experiments and capture data on the eye saccades, fixation duration, and blink times of the participants. The eye movement heat map reflecting the fixation duration of the participants was compared with the CAM generated by machine learning.

### Immediate and Retrospective Verbal Report Methods

In this study, participants used immediate verbal reports to evaluate design images. After the experiment, retrospective verbal reports were used to obtain more information.

## Evaluation Criteria in Design Education

First, we conducted research to obtained answers to question 1: “What are the criteria used to evaluate design education, and how are the weights of each evaluation index allocated?”

### Obtaining Design Evaluation Indicators

Initial Screening of the Indicators for Design Evaluation: After conducting an extensive literature research, we completed a preliminary screening and extracted design evaluation indicators. Some of these indicators are listed in Tables 1-a and 1-b

**Table 1-a.** Indicators Mentioned in the Literature

Literature	Year	Design evaluation indexes
A Study of the Influence of Visual Imagery on Graphic Design Ideation(Simon et al., 2016)	2016	Product Creativity; Practicality; Functionality
Study on Computed Aided Product Decorative Design and Its Evaluation Method(Yang et al., 2008)	2008	Comfortableness; Visual Effect; Modern; Coordination
State-of-the-Art of Design, Evaluation, and Operation Methodologies in Product Service Systems(Qu et al., 2016)	2016	Balancing Customer Value; Sustainability; Perspectives
The Application Research of Fuzzy Mathematics in Design Quality Evaluation of Industrial Product(Liu & Leng, 2019)	2019	Functionality; Structural Elements; Human Factors; Formal Elements; Matching Colors; Environmental Elements
Improvement of Evaluation Method of Elderly Family Medical Product Design Based on AHP(Yue et al., 2022)	2022	Visual Effect; Functionality; Practicality
Research on the Evaluation Index of Product Design based on Consumer, Designer and Manufacturer(Li & Shang, 2013)	2013	Enterprise Planning; User Acceptance; Visual Effect; Brand; Innovation; Trends; Practicality; Development Ability; Cost and Quality Control; Design and Technology
An Online Affordance Evaluation Model for Product Design(Hsiao et al., 2012)	2012	Shape: Reminder, Perception, Appearance, Appropriate Action
		Reactivity: Easy to Operate; Responsiveness
		Clear Information; Symbols; Intuitiveness; Use without Hesitation
Multi-Objective Creative Design Evaluation Method for Industrial Design Cloud Service Platform(Fan et al., 2019)	2019	Practical: Functionality; Reliability; Technical
		Product Creativity: Leading; Value Added; Individuation
		Appearance: Visual Effect; Timeliness; Coordination
		Ecology: Environmental; Economy; Entertainment

**Table 1-b.** Indicators Mentioned in the Literature

literature	Year	Design evaluation indexes
The Evaluation Method and Application Research of Product Design Innovation(Cai & Yang, 2020)	2020	Functionality: Practicality; Safety; Economy; Ergonomics; Product Life
		Visual Effect: Styling; Colors for Matching; Material; Packaging; Workmanship Aesthetics; Spatial Perception
		Experience Design: Semantic Symbols; Emotional Demands; Human–Computer Interaction
		Sustainable Development Design: Product Sociality; Environmentally Friendly; Humanistic Care; Recycling; Universality
		Market Competition: Product Creativity; Market Recognition; Brand Shaping; User Benefits; Industrial Development Prospects
Novelty Evaluation Method of Patent Design Knowledge and Its Application in Creative Design(Qiu et al., 2012)	2012	Product Creativity; Compatibility Degree
Predictive Model of User Experience of Engineering Vehicle Modeling Design Based on Eye Tracking(Lu & Hou, 2017)	2017	Design Integrity; Visual Effect; Emotional Experience
Research and Application of Artificial Intelligent Empowered Design Evaluation Method(YANG et al., 2021)	2021	Visual Effect; Matching Colors; Content
Construction of a Comprehensive Evaluation Index System for Product Innovation Design(Chen & Bi, 2015)	2015	Product Creativity: Unity of Form and Function; Proportional Coordination; Unique Style; Simplicity
		Color Evaluation: Adaptability of Color and Function; Color Quality and Effect; Color Vividness
		Technical Evaluation: Progressiveness; Structural Rationality; Progressiveness of the Technology; Applicability of the Technology; Performance
		Human–Machine Evaluation: Operational Comfort; Good Human–Machine Interface
		Economic Evaluation: Cost; Product Sociality
Research on Multi-Scheme Evaluation System of Product Design based on Industrial Design(WANG, 2021)	2021	Product Sociality; Enterprise Economic Benefits; User Psychology; Development Ability

The tables reveal that evaluation indicators such as available color, practicality, shape, and economy appear more frequently.

**Figure 5.**
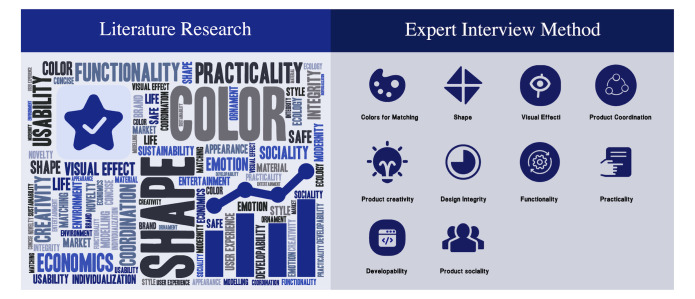
Design Evaluation Indicators

The literature on design evaluation mainly focuses on enterprise or specific products, and there are not many evaluation index systems for design education. To overcome this challenge, we conducted semi-structured interviews with industrial design teachers at Tianjin University and Beijing University of Technology, seeking their input to develop more accurate design evaluation indicators. As a result, we were able to finalize 10 indicators that a suitable for student coursework evaluation. These indicators include colors for matching, shape, visual effect, product coordination, product creativity, design integrity, functionality, practicality, developability, and product sociality, as presented in Figure 5.

Dimension-Reduction Analysis of the Evaluation Indicators Using Factor Analysis: Data were collected from industrial design students and teachers related to design through a questionnaire distributed online. A total of 46 samples were collected, of which four were eliminated as they did not meet the requirements (the questionnaires were completed by non-design related majors), leaving 42 valid samples. Out of these, 34 questionnaires were collected from industrial design and product design majors, which accounted for 73% of the total data. The questionnaires were scored using a 10-point scale. Participants scored 10 design evaluation indicators on a scale of 0 (very unimportant) to 10 (very important) based on a literature search and expert interviews. The data were then subjected to factor analysis using IBM SPSS Statistics 26.

The collected 44 sample data were statistically analyzed, and their mean and standard deviation (S.D.) are shown in Table 2.

**Table 2.** Basic Description of the Data

	Average Value	Standard Deviation
Color	7.45	2.027
Sculpt	7.81	1.941
Visual effect	7.81	1.966
Coordination	8.07	2.041
Creativity	7.26	2.198
Integrity	8.19	1.928
Functionality	8.38	1.780
Practicability	7.71	2.028
Developability	6.83	2.575
Sociality	6.52	2.671

From the table, it can be seen that the average value of functionality is the highest, at 8.38; the lowest, sociality, is 6.52. This reflects that participants generally believe that functionality is more important. The maximum S.D. is for sociality (2.67), and the minimum S.D. is for functionality (1.78).

Explained Variance Ratio: As shown in Table 3, the total variance explained by the five extracted factors after rotation is 86.264%, with each factor having variance interpretation rates of 25.574%, 23.434%, 14.203%, 11.824%, and 11.229%. It is generally recommended that the total variance explained by extracted factors should be above 85% to ensure the more raw data the factor contains. Given that the sum of five factors exceeds this threshold, five factors were extracted in this study.

**Table 3.** Total Variance Explained

	Eigen			% of Variance(Unrotated)			% of Variance(Rotated)		
Factor	Eigen Value	% of Variance	Cumulative% of Variance	Eigen Value	% of Variance	Cumulative% of Variance	Eigen Value	% of Variance	Cumulative% of Variance
1	3.331	33.313	33.313	3.331	33.313	33.313	2.557	25.574	25.574
2	2.428	24.284	57.597	2.428	24.284	57.597	2.343	23.434	49.007
3	1.169	11.686	69.283	1.169	11.686	69.283	1.420	14.204	63.210
4	0.897	8.965	78.249	0.897	8.965	78.249	1.182	11.824	75.035
5	0.802	8.015	86.264	0.802	8.015	86.264	1.123	11.229	86.264
6	0.457	4.566	90.830	-	-	-	-	-	-
7	0.335	3.347	94.177	-	-	-	-	-	-
8	0.258	2.584	96.761	-	-	-	-	-	-
9	0.175	1.753	98.514	-	-	-	-	-	-
10	0.149	1.486	100.000	-	-	-	-	-	-

Factor Loading Table after Rotation: In this study, the data were subjected to varimax rotation to establish the relationship between the factors and research items. Table 4 displays the information extracted from research items and their relationships with the factors. Notably, all research items have a commonality value exceeding 0.4, indicating a strong correlation between factors and research items and effective information extraction by the factors. Factor I encompasses color, shape, visual effects, and product coordination, now referred to as “product visual effects.” Factor II includes functionality, practicality, and developability, and is renamed “product function.” Factor III represents product sociality, whereas Factor IV denotes product creativity. Finally, factor V is dedicated to design integrity.

**Table 4.** Factor Loading (Rotated)

Name	Factor Loading					Communality
	Factor 1	Factor 2	Factor 3	Factor 4	Factor 5	
Colors	0.740	-0.191	0.341	0.219	-0.094	0.758
Shape	0.542	0.018	0.541	0.401	-0.298	0.836
Visual Effect	0.876	0.083	0.111	0.208	0.068	0.834
Coordination	0.908	0.031	0.062	-0.032	0.091	0.838
Creativity	0.196	-0.049	0.014	0.959	0.006	0.961
Integrity	0.058	0.096	0.103	-0.006	0.961	0.947
Functionality	0.082	0.921	-0.007	0.015	-0.102	0.865
Practicability	-0.018	0.862	-0.224	-0.020	0.142	0.824
Developability	-0.106	0.834	0.396	-0.094	0.170	0.902
Sociality	0.254	-0.001	0.876	-0.017	0.172	0.863

Test of Reliability and Validity: When assessing the reliability of a questionnaire, Cronbach's Alpha is a commonly used metric. This value ranges from 0 to 1, with a score below 0.6 indicating insufficient internal consistency. Scores of 0.7–0.8 suggest considerable reliability, whereas scores of 0.8–0.9 indicate very good reliability. In this study, the factor analysis questionnaire achieved a Cronbach's alpha of 0.710, demonstrating good reliability. The reliability table is presented in Table 5.

**Table 5.** Cronbach's Alpha

Cronbach's Alpha	Frequency
.710	10

The questionnaire's validity is determined through the KMO test statistics and Bartlett's test. Table 6 displays that the KMO measure of sampling adequacy is 0.657, falling between 0.6 and 0.7. This indicates that the research data are appropriate for information extraction. Additionally, Bartlett's test of sphericity is 0.000, with a significance level of less than 0.05, making it suitable for factor analysis.

**Table 6.** KMO and Bartlett’s Test

KMO Measure of Sampling Adequacy		.657
Bartlett’s Test of Sphericity	Approx. Chi-Square	182.821
	df	45
	Sig.	.000

### Calculation of the Indicator Weights

Data Collection: We collected data for factor analysis by recycling online data scales from design students and teachers. We obtained a total of 44 sample data, but two samples were removed because they were collected from non-design-related majors. Out of the remaining 42 valid data, 77% of the data were from industrial design and product design majors. After eliminating three pieces of data that failed the consistency check of the matrix, we finally had 39 pieces of valid data. We conducted the AHP using SPSSAU.

**Figure 6.**
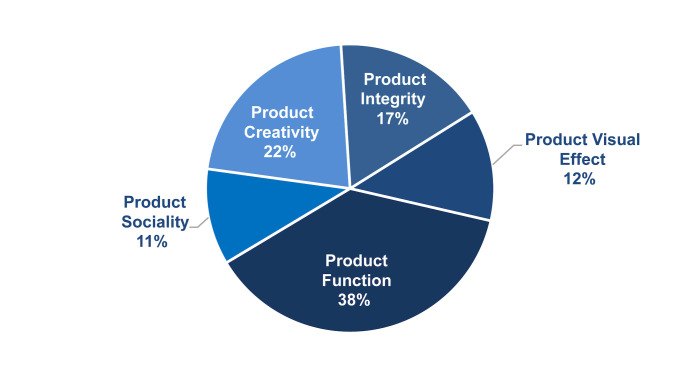
Indicator Weights

AHP Result: Using the AHP, we determined 39 valid data points and assigned an index weight to each one. The average weight of all indicators was then calculated, resulting in a final weight breakdown of 12.48% for product visual effect, 37.81% for product function, 10.78% for product sociality, 21.80% for product creativity, and 17.13% for design integrity. The specific results are displayed in Figure 6.

Reliability Test: Typically, a smaller CR value indicates a more consistent judgment matrix. A CR value below 0.1 confirms that the judgment matrix passes the consistency test, while a CR value above 0.1 suggests inconsistency. In such cases, it is advisable to adjust the matrix and re-evaluate it. To maintain the reliability of the AHP data, any data that fail to meet the consistency test of the judgment matrix are removed prior to calculation.

The design evaluation indicators were initially gathered in this part of the study through an extensive literature review. This was followed by interviews with industrial design teachers to screen the indicators. Data were then collected through questionnaires that were distributed to design industry teachers and students. Through the use of factor analysis and AHP, the evaluation indicators and weight allocation in design education were ultimately established.

## Intelligent Evaluation Model-Based Deep Learning

### Collection of the Image Data

**Figure 7.**
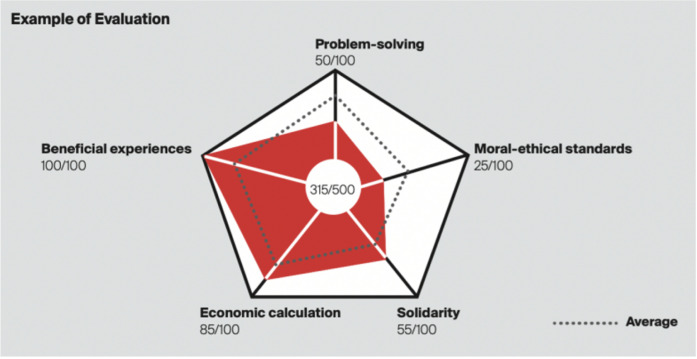
Evaluation Standards for the IF Design Award 2023 Competition

After conducting extensive research and developing evaluation indicators, we discovered that the majority of existing competition evaluation systems align with our indicator systems. As an illustration, Figure 7 (15) demonstrates the evaluation criteria for the IF Student Award 2023, which includes problem-solving aspects such as innovation, refinement, uniqueness, usage value, and usability. Using the evaluation indicators that we established, we gathered the winning entries from competitions that employ similar criteria and categorized them according to their award level. Our ultimate goal was to use deep learning techniques to automatically evaluate product design work.

We collected a total of 33,745 award-winning works from various design competitions such as the Red Dot Award, IF Design Award, and IDEA since 2015.

Our selection process focused on a variety of image display types, including product angle, background complexity, and human–machine displays, to create a more comprehensive deep-learning data set.

To avoid confusion, we selected the highest level award for repeated products and included them in the data set during the data compilation stage. Additionally, we ensured that only one work of the same level was selected once. This is important because some designs may have won both international and national awards. Furthermore, because some design competitions are not only focused on product design, but also include graphic design, interactive interface design, spatial display design, and other content, we removed them when screening images to ensure the accuracy of the dataset and reduce interference with the model.

We classified all award-winning works into international (Lv. 1), national (Lv. 2), and provincial (Lv. 3) levels. The gold and silver awards for each level were classified as excellent (01), whereas the bronze and excellent awards were classified as ordinary (02). Among them, there were 2,449 design images for Lv. 1 01, 26,729 design images for Lv. 1 02, 433 design images for Lv. 2 01, 2,082 design images for Lv. 2 02, 322 design images for Lv. 3 01, and 1,730 design images for Lv. 3 02.

### Deep Learning Neural Network Computation

In deep learning, CNN exhibits advantages in image processing due to its unique structure and characteristics. Through structures such as convolutional layers and pooling layers, CNN can effectively extract useful features from original images. These features not only include low-level edge and texture information but also higher-level semantic information. Additionally, CNN possesses characteristics of weight sharing and local connectivity, which allows the model to utilize parameters more efficiently, reduce computation, and enhance the model's generalization ability when processing images. Among convolutional neural network models, VGG16 is widely adopted due to its excellent performance and relatively simple structure. In this study, the VGG16 is used for model training. Deep learning was conducted on the award-winning images in the design competition using a 16-layer VGG network.

This deep neural network comprises 16 layers, which include 13 convolutional layers and three fully connected layers, with pooling operations that double the processed data dimensions. During training, an RGB image with a size of 224 × 224 is inputted, which means the input image size is 224 × 224 × 3. After five sets of convolution and pooling operations, the final output measures 7 × 7 × 512. Following this, two fully connected layers consisting of 4,096 channels each and one fully connected layer with 1,000 channels for classification are connected, and the final result is obtained through a softmax activation function.

**Figure 8.**
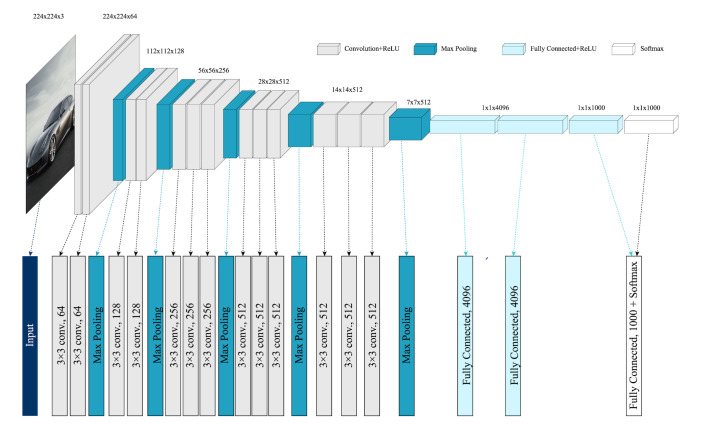
Architecture of the VGG16

During the in-depth study, we found that the number of award-winning entries at the excellent level was less than that of the ordinary ones. To address the issue of imbalanced categories and enhance the precision of our computations, we implemented focal loss. The focal loss function was proposed in 2017 by He Kaiming et al. in the paper “Focal Loss for Dense Object Detection” (23) to address the imbalance between positive and negative samples and the difficulty of learning difficult samples in one-stage object detection in the field of image processing. This loss function differentiates between easily classifiable and difficult-to-classify samples, placing greater emphasis on the latter while reducing the weight of the former. The formula for focal loss is as follows:


$$FL=-\sum_{i=1}^{N}{\left(1-P_{i}\right)}^{\gamma }\log\left(P_{i}\right)$$

The data set of each layer was divided into a training set, verification set, and test set according to the ratio of 8:1:1.

The accuracy results of the model for the international, national, and provincial and ministerial award-winning works are 72.96%, 68.65%, and 67.48%, respectively. In previous studies, the accuracy of the VGG model was over 60% (46). Compared with the accuracy of other VGG models in design classification, the accuracy of this model on product design images is effective.

Upon conducting research in this area, it was discovered that by providing a substantial number of images of winning designs in a design contest, the implementation of a deep learning neural network can effectively categorize and assess the product images with good accuracy. This can facilitate the intelligent evaluation of product design images.

## Correlation in Focus and the Difference between Human Evaluation and Machine Evaluation

Section 5 revealed that deep learning neural networks can intelligently evaluate design images. Human evaluation is typically based on experience and knowledge. It is worth exploring whether there is a connection between the neural network's working principles and human subjective evaluation. To investigate, an eye movement experiment was conducted to generate a heat map of the fixation duration and it was compared with the CAM of the CNN.

### CAM Drawing and Analysis

The CAM (Class Activation Mapping) is a visualization technique that displays the weight or center of gravity of a model during training and which part of the image the classification model uses for judgement(47). This technology aids in understanding how CNNs make classification decisions by revealing the areas within an image that are most significant for the CNN in determining a particular category.

**Figure 9.**
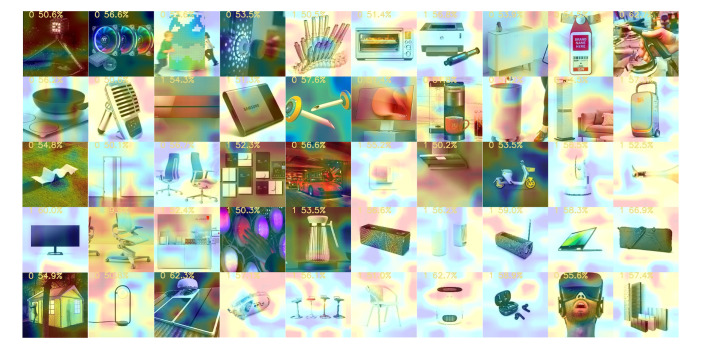
CAM Output by the CNN

The method involves generating a heat map by integrating the feature map's weight. The process uses a new CNN, from the input image to the CNN, and then to a global average pooling layer. Five-hundred and twelve average values can be obtained from the last global average pooling layer, and after 512 features have been obtained, the final linear layer is used to let the neural network learn which weight is greater for the obtained 512 features and finally output the prediction result. Figure 9 depicts a part of the CAM process.

Through the study of the CAM, we obtained the following findings:

(1) The CNN's focus is not entirely on the product, as we initially thought. More often, the focus is on the background, which is more obvious when the product has a white background.

(2) When the product color is dark on a light background, the CNN focuses on areas of color change, such as product edges or light and shadow shifts.

(3) For light products, it is easier for the CNN to focus on the background when the background is light.

(4) In the case of a complex background, the CNN algorithm tends to focus more on the darker areas of the image, such as the shadow beneath the wheel hub.

(5) Additionally, the corners of the image are also more likely to be noticed by the algorithm.

### Gathering of Eye Tracking Data

Eye-tracking research was first used in reading studies, but it has become increasingly popular in sociology, psychology, and other research fields. First, we design an experimental Design of Eye-Tracking

**Figure 10.**
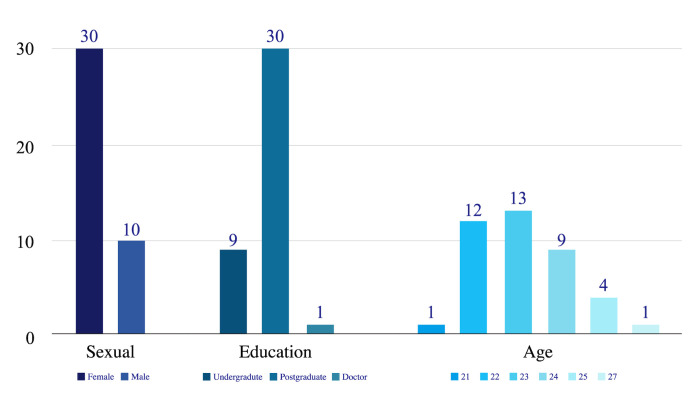
Basic Information Distribution of Participants

Participants: A total of 40 participants (10 men, 30 women) aged 21–27 years were recruited to participate in the eye movement experimental research. All of the participants were master’s, doctoral, or undergraduate students of three degrees or more in industrial or product design with a minimum of three years of design experience. The basic information distribution of participants is shown in Figure 10. To ensure the precision of the eye movement data, it was communicated to the participants during recruitment that they should not have high myopia or astigmatism, and they must possess normal or corrected-to-normal vision and not have any underlying eye-related ailments. Subjects were asked to wear glasses with an appropriate prescription, and contact lenses were not allowed. Before the experiment, each participant read and agreed to the informed consent form. We recruited a total of 40 subjects. Because of the limitations of the tracking box size, the subjects' data were lost when they swung their heads over a large range. Therefore, after the experiment, we carried out data verification and reserved the data with an eye movement data loss rate of less than 15%. This resulted in a total of 34 samples of valid data: 10 males (M=23.20,SD=1.30) and 24 females (M=23.13,SD=1.19).

During the expert interview stage, the researchers conducted interviews with design teachers at universities to identify the most common product types used in their assignments. We also asked them about the types of product design that students are more likely to choose when given open-ended topics.

**Figure 11.**
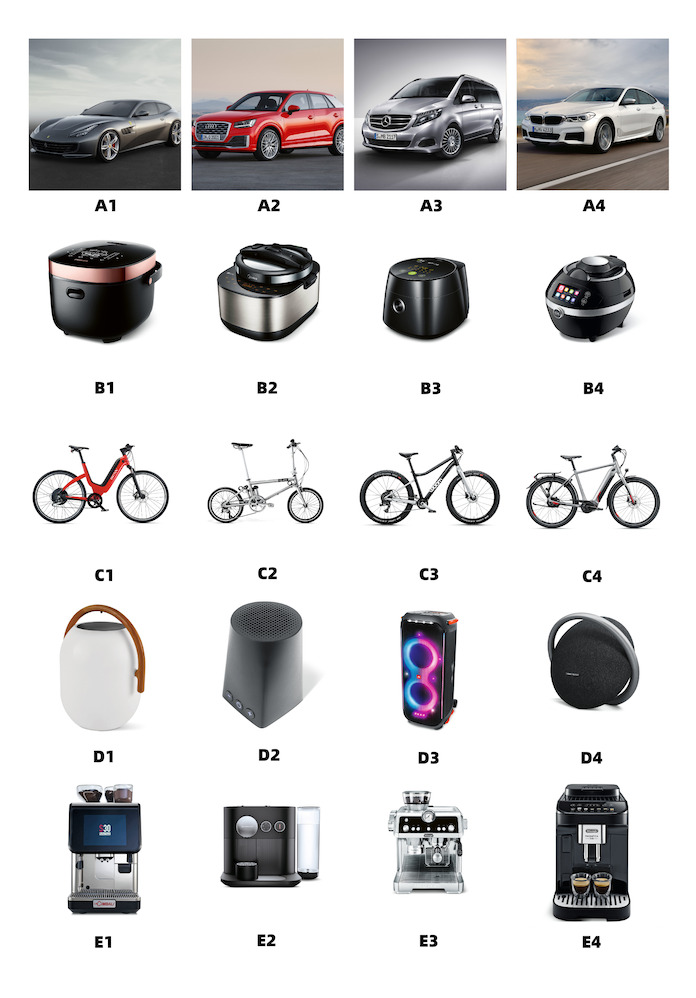
Stimulus Materials

Moreover, considering the types of graduation projects in recent years and categories of award-winning products in competitions, we selected five design product categories from the 33,745 images for the study. These categories included cars, rice cookers, bicycles, loudspeakers, and coffee machines, which were named Groups A–E. A total of 20 design product pictures were selected from 4 production pictures per category as stimulus materials, named A1-A4, B1-B4, C1-C4, D1-D4, E1-E4. To ensure the accuracy of the test and minimize interference, we used the same angles for each group of images during the screening process. This included the three most common product display angles: perspective view, front view, and left view. We selected four product images for each type, resulting in a total of 20 design product images as stimulus materials. The background was kept consistently white, except for Group A images. Figure 11 presents a visual representation of the stimulus materials.

**Figure 12.**
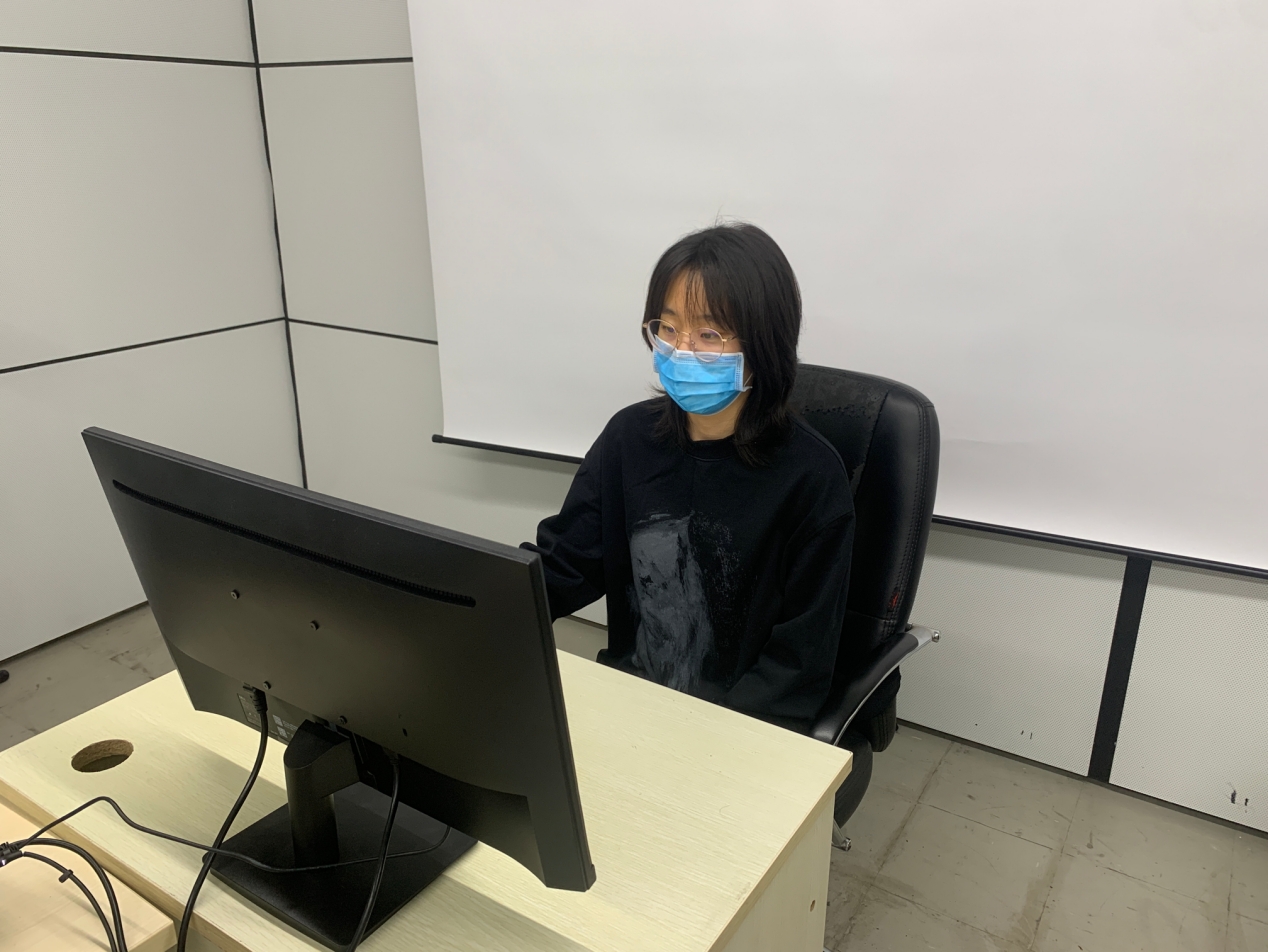
Experimental Environment

**Figure 13.**
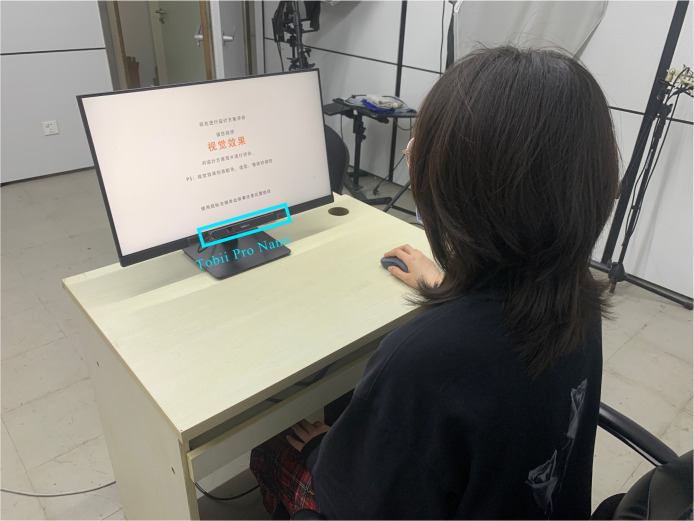
Operating Environment of the Equipment

Experimental Environment and Equipment: To reduce the impact of external factors such as light changes, noise, and human influence, the experiment was conducted in a room without windows. To ensure comfort and precision, an adjustable seat was used to assist with positioning the subject and eye tracker. The Tobii Pro Nano, a lightweight and compact eye tracker, was chosen for this experiment. It easily connects to a host device via USB and can be installed on either a laptop or an external monitor. This screen eye tracker is less obtrusive and less invasive than the glasses eye tracker, making it more comfortable for the subject. A Dell 24-inch external display screen was used in the experiment, and the resolution was 1920 × 1080 (16:9). The experimental host was a Dell XPS15. The experimental environment is shown in Figure 12, The operating environment of the equipment is shown in the Figure 13.

Experimental Process: This process involved preparing materials and calibrating equipment. It was crucial to inform the participants about the experiment's purpose and specific requirements.

Next, Experiment I was conducted. The subjects assessed the overall product design scheme. Four product design schemes were presented on a page, and the participants shared their rating results with the experimenter. There was no time constraint for this session, and the subjects carefully observed the four design schemes on the screen.

After all groups were scored, the indicators were evaluated and Experiment II was conducted. Participants were informed of the evaluation index before the assessment began, and they evaluated the product design images as per the index. To avoid memory bias, the subjects first viewed the design images for 14 s and then evaluated four design images on the screen simultaneously. Before the image appeared, a fixation point of 500 ms was presented to remind the subjects to concentrate their attention, and the starting point of each subject's gaze was the same. To prevent exhaustion, participants were given a rest period of 15 s or more after each evaluation.

Finally, an interview was conducted with the subjects after the experiment to gather further information.

**Figure 14.**
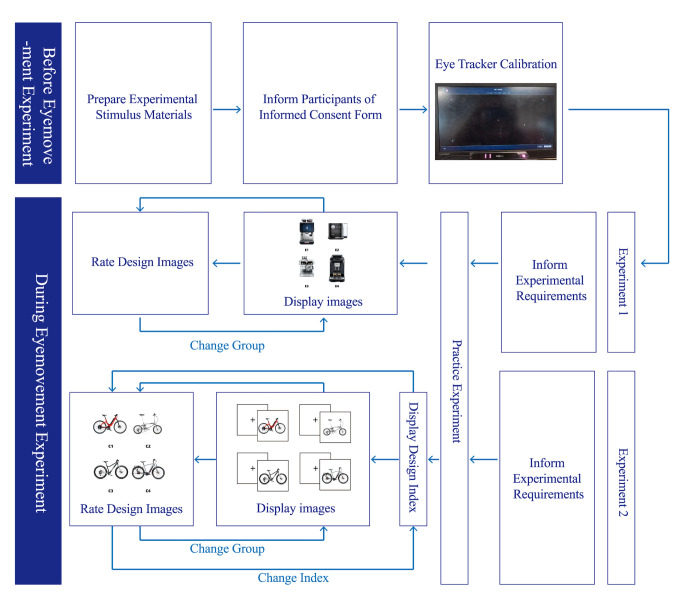
Experimental Process

To account for the limited number of subjects, an in-subject design was used for this experiment. To avoid potential carryover effects that could arise from repeated exposure to the same images, the order of stimuli presentation was altered for each participant. The study employed a partial counterbalancing–balanced Latin square design methodology. Furthermore, the experimental group was provided with practice opportunities before the commencement of both experiments to ensure familiarity with the process. A visual representation of the experimental process can be found in Figure 14.

### Analysis of Eye Tracking Data

**Figure 15.**
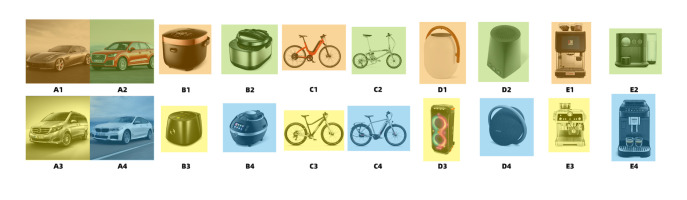
AOIs of the Image Stimuli

Data Screening and Processing: During data cleansing, median filtering was used to filter out noise from the eye movement data. Additionally, any gaze shorter than 60 ms was removed. In the evaluation and scoring page of Experiment 1, we divided the images of different products into different areas of interest (AOIs). As shown in Figure 15, different colors represent different AOIs. We extracted the data of different AOIs separately to obtain the eye movement data of the participants in the product image area during evaluation. We collected four groups of data (AOI visits, AOI fixation times, AOI fixation duration, and AOI total access duration) for subsequent analysis.

**Figure 16.**
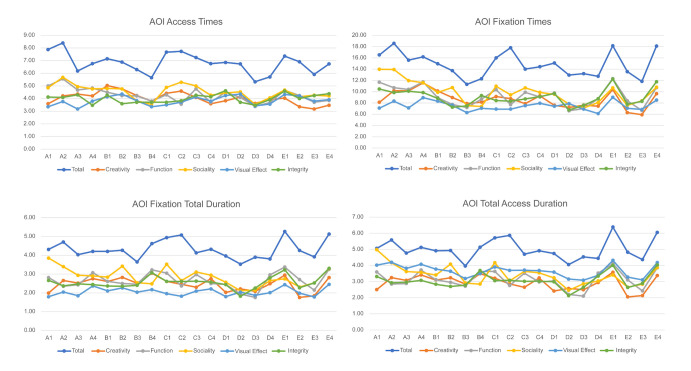
Line Chart of Eye Movement Data

**Figure 17.**
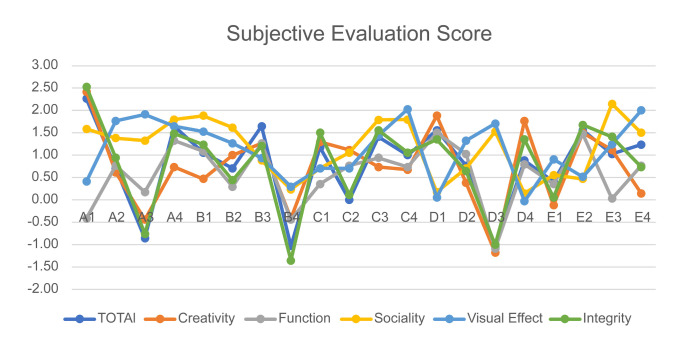
Subjective Evaluation Score

**Figure 18.**
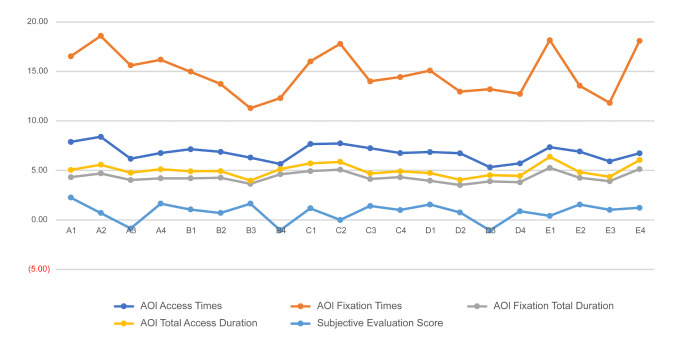
Subjective Evaluation Score and eye movement data

During the eye movement experiment, ErgoLAB v3.17.2 was used to collect the AOI access times, AOI fixation times, AOI fixation total durations, and AOI total access durations of the 34 participants. The average values are shown in Tables 7 and 8. Line charts of the four types of data are shown in Figure 16, and the subjective evaluation scores are shown in Figure 17. To explore the relationship between the eye movement data and subjective evaluation data, the scores of all types of eye movement data and the subjective evaluation scores were plotted as a line chart (Figure 18), and the correlation was explored through the trends of the lines.

**Table 7.** AOI access times and AOI fixation times

	AOI access times						AOI fixation times					
	Total	Creativity	Function	Sociality	Visual Effect	Integrity	Total	Creativity	Function	Sociality	Visual Effect	Integrity
A1	7.88	3.59	5.00	4.85	3.35	4.12	16.52	8.12	11.68	13.97	7.09	10.47
A2	8.39	4.21	5.56	5.68	3.76	4.09	18.58	10.18	10.71	13.94	8.32	9.85
A3	6.18	4.35	4.68	4.94	3.18	4.29	15.61	10.15	10.41	11.97	7.12	10.09
A4	6.76	4.21	4.82	4.76	3.79	3.47	16.18	11.56	11.74	11.59	8.94	9.85
B1	7.15	5.03	4.50	4.79	4.15	4.26	14.97	10.12	8.94	9.88	8.32	8.82
B2	6.88	4.76	4.24	4.76	4.35	3.59	13.74	8.97	7.74	10.74	7.59	7.24
B3	6.29	4.24	4.21	3.82	3.82	3.71	11.29	7.91	7.21	7.53	6.29	7.47
B4	5.65	3.79	3.79	3.53	3.35	3.71	12.29	8.15	8.68	7.38	7.06	9.32
C1	7.67	4.41	4.29	4.88	3.50	3.71	16.00	9.15	10.38	10.97	6.91	8.44
C2	7.73	4.59	3.56	5.29	3.71	3.82	17.79	8.76	7.71	9.44	6.91	8.32
C3	7.24	4.09	4.76	5.00	4.12	4.26	14.00	7.88	9.88	10.68	7.53	8.71
C4	6.76	3.59	3.71	4.26	3.79	4.15	14.42	9.26	9.06	9.88	7.94	9.12
D1	6.85	3.82	4.50	4.44	4.24	4.62	15.08	7.59	9.76	9.53	7.41	9.68
D2	6.74	4.12	4.09	4.53	4.35	3.71	12.95	7.21	6.62	7.79	7.88	6.76
D3	5.32	3.62	3.38	3.53	3.41	3.47	13.20	7.62	7.00	7.12	6.91	7.59
D4	5.71	3.91	3.68	4.06	3.56	3.91	12.74	7.44	8.76	8.09	6.12	8.74
E1	7.35	4.03	4.65	4.68	4.32	4.59	18.15	10.38	12.32	10.68	9.00	12.24
E2	6.91	3.35	4.24	4.15	4.21	4.00	13.56	6.29	8.38	7.59	7.06	7.79
E3	5.91	3.18	3.71	4.26	3.79	4.24	11.82	5.91	6.68	8.38	6.85	8.29
E4	6.74	3.47	3.85	4.21	3.91	4.38	18.09	9.65	10.74	10.79	8.50	11.76

**Table 8.** AOI fixation total duration and AOI total access duration

	AOI fixation total duration						AOI total access duration					
	Total	Creativity	Function	Sociality	Visual Effect	Integrity	Total	Creativity	Function	Sociality	Visual Effect	Integrity
A1	4.32	1.99	2.82	3.85	1.79	2.67	5.06	2.50	3.60	4.98	5.92	3.31
A2	4.70	2.66	2.36	3.40	2.05	2.37	5.58	3.25	2.85	4.17	6.21	2.93
A3	4.03	2.52	2.43	2.94	1.85	2.47	4.77	3.08	2.88	3.63	5.62	2.97
A4	4.21	2.76	3.07	2.90	2.37	2.44	5.12	3.41	3.72	3.57	5.97	3.08
B1	4.21	2.61	2.61	2.84	2.11	2.37	4.91	3.10	3.12	3.41	5.56	2.84
B2	4.27	2.82	2.50	3.42	2.27	2.35	4.93	3.24	2.96	4.08	5.34	2.70
B3	3.65	2.52	2.46	2.54	2.04	2.40	3.97	2.84	2.72	2.91	4.63	2.78
B4	4.62	3.05	3.23	2.48	2.18	3.07	5.14	3.49	3.63	2.84	5.02	3.69
C1	4.94	2.61	3.05	3.52	1.96	2.61	5.71	3.21	3.63	4.17	5.72	3.06
C2	5.07	2.46	2.37	2.65	1.82	2.61	5.86	2.86	2.76	3.04	5.42	3.08
C3	4.13	2.30	2.97	3.11	2.10	2.62	4.70	2.65	3.52	3.63	5.45	3.01
C4	4.32	2.76	2.50	2.94	2.21	2.57	4.91	3.22	2.98	3.55	5.36	3.02
D1	3.96	2.02	2.45	2.57	1.80	2.44	4.74	2.42	3.05	3.23	5.28	2.99
D2	3.53	2.21	1.93	2.10	2.04	1.82	4.06	2.58	2.21	2.46	4.62	2.13
D3	3.90	2.08	1.76	2.15	1.87	2.26	4.53	2.50	2.10	2.87	4.46	2.65
D4	3.81	2.49	2.96	2.65	2.01	2.80	4.44	2.95	3.52	3.04	4.88	3.34
E1	5.27	2.94	3.37	2.75	2.44	3.22	6.38	3.58	4.11	3.41	6.36	4.01
E2	4.26	1.76	2.71	2.31	1.98	2.27	4.83	2.05	3.11	2.66	4.79	2.63
E3	3.92	1.84	2.15	2.53	1.79	2.53	4.36	2.14	2.42	2.84	4.52	2.89
E4	5.13	2.82	3.25	3.29	2.46	3.31	6.05	3.37	3.85	3.87	6.07	4.03

Analyzing the data and line chart, we draw the following conclusions:

(1) The data presented in the line chart indicates that participants exhibit a higher degree of attentiveness during the overall evaluation than during the evaluation of a specific index. This observation is supported by the eye movement data, which unequivocally demonstrate the participants' increased focus on the overall evaluation. During the total evaluation, the participants' AOI access times, AOI fixation times, AOI fixation duration, and AOI total access duration indicators were significantly higher. The interviews conducted with the participants revealed that during the overall evaluation, the subjects scanned the image multiple times according to the evaluation index, thereby increasing the total number of scans. This phenomenon highlights the importance of considering multiple evaluation indicators to make a comprehensive and accurate assessment. It provides valuable insights into evaluation behavior and can guide us in optimizing evaluation methods.

(2) According to the findings of the study, participants displayed a higher degree of tolerance for the sociality index while evaluating various indicators. They claim that the rationale behind this behavior is the lower weight assigned to sociality factors than to other indicators, which consequently warrants a less stringent assessment.

(3) On the basis of the subjective evaluation scores provided by the study's participants, it is evident that the trend of scoring for each evaluation index is in alignment with the overall product score. Furthermore, a statistically significant positive correlation has been observed between the aforementioned variables. Especially in terms of creativity and integrity, this correlation is obvious. These findings suggest a strong association between the evaluation indices and the overall product evaluation, highlighting the importance of considering multiple facets in product assessment and further demonstrating the effectiveness and stability of these indexes in evaluating the product.

(4) Analysis of the eye movement data reveals that, regardless of the type of eye movement data considered in this study, there is a consistent trend in the evaluation of each index and the overall evaluation of the product. This is evident from the line graph because it shows a consistent trend.

(5) A comparison of the eye movement data and scores of participants' subjective evaluation indicates that the scoring results are consistent with the trends in the eye movement data. Although the scoring results do not perfectly align with the recorded eye movement data, they show a similar trend. Upon conducting additional interviews, it was revealed that participants tended to increase their viewing times by comparing two products that received similar scores. This behavior did not necessarily correspond with whether the final score was high or low.

**Figure 19.**
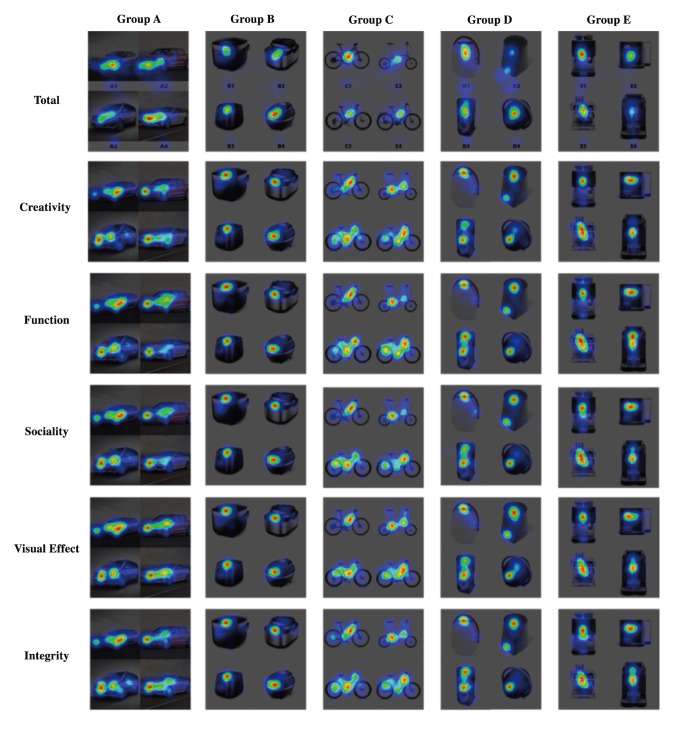
Synthesized Eye-Tracking Heat Map

**Figure 20.**
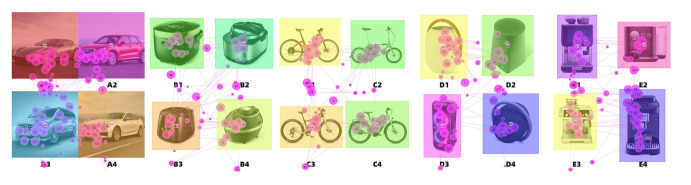
Track Map of Participant 1

The eye movement data collected in Experiment 2 included various information, such as pupil data, fixation duration, gaze point, saccade data, and fixation coordinates. ErgoLAB v3.17.2, the software used for data acquisition and analysis, was used to generate a heat map and eye movement track map for one participant. These heat maps allow us to gain a more direct understanding of the subject’s focus. However, the software cannot directly generate an eye movement heat map for the eye movement data of 34 participants. Hence, we created eye movement heat maps for 34 data sets by synthesizing the coordinate position information and fixation duration. The resulting thermogram of eye movement is displayed in Figure 19. By contrast, the eye movement trajectory diagram reveals each subject’s observation sequences during saccades. The eye movement pattern of subject 1 in Experiment 1 is shown in Figure 20.

By studying the eye-movement heat maps and track maps, we obtained the following findings:

(1) During the evaluation of various design indicators in the images, the viewing position of the same product design image was fixed. However, subtle differences were noticed, such as a larger gaze range when the index was integrity. However, in Experiment I, the center of the image remained the primary focus during the overall evaluation process.

(2) When evaluating four images on a page, observers make more comparative observations on of the pictures with close grades that people feel hesitant to determine their final ranking.

(3) For Group A (cars), it was found that the majority of the participant attention was drawn to the car's brand logo and headlights, while the rear of the vehicle was often overlooked. Interviews revealed that the headlights in the perspective view had a more prominent visual impact, leading subjects to focus on them more. Furthermore, because a car is a complex product, the subjects needed to perform more scanning to obtain more information about the product.

(4) When evaluating Group B (rice cooker) images, the subjects mainly focused on the operation panel. Some subjects mentioned in the interview that they would look for a longer time because they were curious about the content on the panel.

(5) When observing Group C (bicycle) images, subjects focused their attention on the main parts of the bicycle frame, such as the top tube, head tube, and pedal. Compared with folding bicycles, mountain bikes have more viewing areas.

(6) Group D (loudspeakers), as a product with a relatively simple appearance and shape, less attention was paid to the critical points in the evaluation, and the participants mainly focused on the details such as buttons and labels.

(7) In the evaluation of Group E (coffee machines), subjects paid more attention to the position of the operation panel, button, and group head. In the functional evaluation index, the group head received more attention.

### Comparative Analysis of Intelligent Evaluation Data and Eye-Tracking Data

A CAM is a powerful tool that can give researchers a more intuitive and accurate analyses of the focus of neural networks in design evaluation. The colors on a CAM indicate the attention levels, with red indicating high attention and blue indicating low attention, which aligns with the eye movement heat map. Thus, by examining the color distribution on both the CAM and heat map, we can assess the similarities and differences of the two in design evaluation.

**Figure 21.**
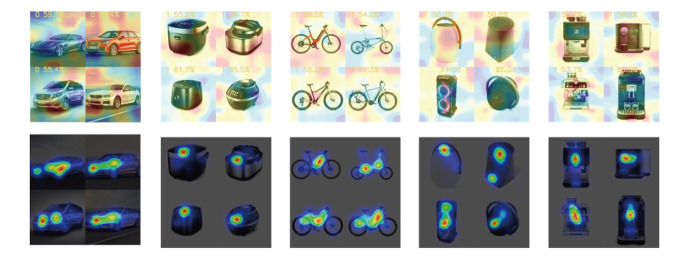
Eye-Tracking Heat Maps and CAMs of the CNN

Through a comparison of the eye movement heat map and the CNN CAM, we discovered that machine learning and human evaluation share similar focus regions. For example, in the car group, A4 eye tracking and deep learning both prioritize the front windshield, whereas in the rice cooker group, B2 the operation panel is emphasized. For the bicycle group, C3, attention is directed towards the bicycle frame. In the audio group, D2 and D4 share a similar focus, with D2 honing in on the top of the fade-out surface and D4 focusing on the bottom logo. For the coffee set group, E2 and E3 demonstrate a strong correlation and focus on positions such as the knob and operating panel, whereas E4 displays a weaker correlation to the panel position. When examining the results for the camera group, it can be found that in the assessment of the design using deep learning, there is a greater emphasis on the product itself, particularly on the parts displaying prominent changes in color. The eye-tracking heat map and CAMs of the CNN are shown in Figure 21.

An examination of the figure reveals that there are some differences in the focus of the eye movement heat map and deep learning CAM output. The CAM shows that the model may concentrate on certain elements outside the product, such as the background, despite the absence of actual content in those areas. However, humans seldom pay attention to the background and instead tend to disregard it when rendering subjective evaluations. Despite the presence of background content, the human focus tends to be primarily directed toward the product itself, which also leads to the difference in the focus between human eyes and the deep learning model.

Since eye movement experiments use images as evaluation materials, and images themselves are composed of basic low-level feature elements, including color, contrast, foreground and background, etc. Therefore, advanced evaluation features are inevitably related to these basic elements. In the experiment, when we asked the subjects to evaluate, users had a certain amount of time to think. Therefore, the gaze results are not limited to focusing on superficial features, which have less impact on users. These superficial features are not discussed in this study. However, in the future, the degree of influence can be determined through comparative experiments, making the results of this study more rigorous.

Note that while neural networks exhibit certain resemblances to human cognitive processes, as an algorithm, they do not represent a complete mirror image of human behavior. In the realm of artificial intelligence, traditional neural network models mainly simulate the connections and information transmission mechanisms between neurons in the human brain to learn and predict input data. Hence, despite the presence of certain parallels with human thought processes in the model, it is a mathematical model at its core, and there are still many differences that deserve further research and exploration.

## Conclusion and Discussion

Our study aimed to establish an evaluation index system for design education and create an intelligent evaluation system. We conducted an in-depth analysis of the correlation between eye movement and subjective evaluation, as well as an eye movement heat map and CAM. By comparing the differences and similarities between human-based and computer-based design evaluation, we were able to draw valuable insights. Our research yielded the following findings:

Through interviews, literature reading, factor analysis, and an AHP, we established an evaluation index system suitable for student product design coursework.

By conducting interviews, reviewing relevant literature, performing factor analysis, and using the AHP, we were able to develop a weighted comprehensive evaluation index system: 12.48% for product visual effect, 37.81% for product function, 10.78% for product sociality, 21.80% for product creativity, and 17.13% for design integrity. This evaluation system allowed students to more intuitively understand the strengths and weaknesses of their product designs based on the teacher's scoring, enabling them to make more targeted modifications.

The VGG16 model is proficient at classifying and predicting design product images, making it a good tool for automated product design work evaluation. Its evaluation outcomes are accurate, offering an effective means for enhancing evaluation efficiency and providing a valuable reference for evaluation in design education.

An analysis of the statistical data gathered from participants' subjective evaluation scores revealed that a positive correlation exists between the evaluation scores of each index and the overall product evaluation results within the design evaluation process. This suggests that each index accurately reflects the product's overall evaluation to a certain extent, thus validating the effectiveness of the design evaluation indices for design education-focused assessments. These findings provide a scientific basis and valuable reference for future design evaluations.

The analysis of the eye movement data indicates the following: Participants will conduct multiple reviews based on different indicators during the overall evaluation to obtain the overall evaluation results. In addition, there is consistency in the trends of the eye movement data for the overall evaluation and the individual evaluation of each indicator. The subjective scoring is not always consistent with the eye tracking data, which can be influenced by similar-rated products. Eye tracking heatmaps reveal that individuals tend to pay more attention to the product itself rather than its background during the subjective evaluations, with the functional areas of the product receiving particular focus.

Upon analyzing the CAMs, it can be inferred that the neural network learning process does not solely prioritize the product itself but also pays heed to a part of the background. The comparison of the eye-movement heat map and CAM showed that there was a correlation between the emphasis on human-based evaluation and computer-based evaluation. When conducting design evaluations, people and AIs mostly focus on the product itself. However, an AI will place some of its focus on the background. In areas where the product form and color have undergone significant changes, CNN models pay more attention. When people evaluate a design, they have a higher interest in the functional-related locations.

After conducting a series of studies, we developed a reliable design evaluation system that enables teachers to assess their students' design coursework more objectively. Additionally, we have created an efficient intelligent automatic design evaluation model that meets our expectations in terms of accuracy. It can help design teachers to make more objective and accurate judgments when evaluating a student’s product design assignment, further improving the efficiency of design education evaluation.

Moving forward, it would be possible to delve deeper into design assessment by incorporating physiological signals such as EEG and eye movement to better understand the connection between design evaluation and subjective perception. The automatic evaluation model could also be refined to enhance prediction accuracy, aligning with the sub-item design evaluation index system. This would provide further exploration for automatic evaluation and automatic feedback in the future. Ultimately, the aim of these efforts is to enhance the quality of student design work and bolster the evaluation system in design education.

### Ethics and Conflict of Interest

The author(s) declare(s) that the contents of the article are in agreement with the ethics described in http://biblio.unibe.ch/portale/elibrary/BOP/jemr/ethics.html and that there is no conflict of interest regarding the publication of this paper.

### Acknowledgements

We would like to express our gratitude to the designers and participants for their contributions to the current study. This study was supported by the National Natural Science Foundation of China (62277038).
